# SignBridge Bilingual Sign Language Avatar—Construction Principles and Experts Quality Assessment

**DOI:** 10.3390/s26123642

**Published:** 2026-06-07

**Authors:** Nurzada Amangeldy, Marek Milosz, Aigerim Yerimbetova, Nazira Tursynova, Bekbolat Kurmetbek, Nazerke Gazizova

**Affiliations:** 1 Institute of Information and Computational Technologies of the Committee of Science of the Ministry of Science and Higher Education of the Republic of Kazakhstan, Almaty 050000, Kazakhstan; amangeldi_n_3@enu.kz (N.A.); ntursynova000@gmail.com (N.T.); b.kurmetbek@kmge.kz (B.K.); g.nazerke2755@gmail.com (N.G.); 2 Faculty of Information Technologies, L.N. Gumilyov Eurasian National University, Astana 010008, Kazakhstan; 3 School of Engineering and Information Technology, META University, Almaty 050012, Kazakhstan; 4 Department of Computer Science, Lublin University of Technology, 20-618 Lublin, Poland; 5 SignBridge LLP, Astana 010000, Kazakhstan

**Keywords:** multilingualism, sign language, avatar, quality evaluation

## Abstract

The multilingualism found in many countries, as well as within professional groups, complicates verbal communication, as both communicating parties are required to know all the languages used. This problem is exacerbated by the fact that languages are often mixed during communication. Avatars can be used to communicate with deaf people by simulating the behavior of sign language users. This paper presents a digital sign language avatar built on a language-agnostic, multimodal animation pipeline that decouples linguistic input from animation, combining skeletal body and hand motion with facial blendshape animation as independent modalities. It also presents a methodology for assessing its quality with the participation of experts (i.e., professional sign language interpreters) and the corresponding research results. The average quality rating of the avatar interface by the experts was 5.5 on a 7-point Likert scale, indicating its potential for practical use. At the same time, the research identified opportunities to improve the naturalness of movement and the consistency of gesture transitions.

## 1. Introduction

The phenomenon of bilingualism and multilingualism has become increasingly prevalent in many countries due to a number of factors, including linguistic globalization, migration processes, and the rapid development of digital communication. According to data from UNESCO, the global number of languages exceeds 7000, with a substantial proportion of the population utilizing multiple languages in their daily interactions [1]. This phenomenon gives rise to the interconnection, interaction and transformation of languages, thereby increasing the demands on digital tools that facilitate communication, particularly on interfaces that interact directly with the user. This phenomenon occurs primarily in two different contexts: territorial and professional.

The first context of multilingualism, territorial, is the result of population mixing. These processes resulted from natural migration, but most often they are the result of historical and socio-political processes. For instance, in the United States, official statistics demonstrate that more than 350–430 languages are in use (included: English, Spanish, and French), thus indicating that bilingualism and multilingualism are the norm in the country’s linguistic environment [2]. In everyday interactions, English and French are mixed (e.g., in Louisiana) or English and Spanish (California). A similar situation occurs in many other countries. Examples include Switzerland (three languages in daily use) and Tunisia (a mixture of Arabic and French). In the post-Soviet space, the linguistic situation is characterized by its own unique historical peculiarities. The language policy implemented during the Soviet era influenced the development of many national languages, resulting in the emergence of linguistic asymmetry and functional restrictions. The development of sign languages alongside spoken and written languages has been influenced by these processes. Consequently, within the prevailing digital milieu, certain languages have attained a dominant status, while local sign languages were often marginalized, partially assimilated, or insufficiently documented, leading to blurred linguistic boundaries and reduced institutional recognition.

In the labor market, bilingualism and multilingualism are often considered professional requirements. Consequently, in sectors such as education, healthcare, public administration, banking, and transportation, effective communication with users speaking different languages is essential. In some professions (e.g., IT), multilingualism is common, also in terms of internationalization and remote work.

As stated by the World Health Organization, more than 430 million people worldwide are affected by hearing loss, with sign languages being autonomous, natural languages with their own complex grammatical and syntactic structures, serving as the primary means of communication for the Deaf Community [3]. It is imperative to recognize the significance of facilitating access to information for deaf users.

Digital avatars play a significant role in improving information accessibility. This includes communication with deaf users. They play a particularly important role because they facilitate the visualization of text or spoken information using sign language, thus serving as the primary communication channel for hearing-impaired users [4]. Therefore, it is crucial to treat avatars not only as visual images but also as interactive interface components that facilitate social and communicative interaction with the user [5].

Sign language avatars are linguistically complex systems that must accurately convey information, taking into account grammar, spatial organization, and non-manual elements of sign language (facial expressions, head movements) [6]. In such systems, the avatar acts as a communicative agent rather than a conventional graphical user interface element, serving as an intermediary between the digital environment and the sign language user.

The multilingual nature of the verbal message rendered in sign language by a digital avatar poses a significant challenge, complicating the process of creating it and potentially reducing its quality. Therefore, assessing its quality assessment becomes a critical task.

From the recipient’s point of view, digital sign language avatars can be considered a specific type of graphical user interface (GUI) of information systems. According to the ISO 9241 standard, GUI quality is determined by users achieving their goals with efficiency, effectiveness, and satisfaction [7]. In the Nielsen usability model, these criteria are supplemented with indicators such as ease of learning, memorability, and the number of errors [8].

The purpose of this paper is to present SignBridge Avatar built on a language-agnostic, multimodal animation pipeline. The system decouples the linguistic input from the animation layer: any gloss sequence, regardless of the source language pair, is mapped to a unified animation representation combining two independent modalities—skeletal body and hand motion, and facial blendshape animation for lip articulation and non-manual expression. This design allows the system to support any language pair without structural modifications and the present study it is demonstrated for the Kazakh-Russian bilingual context.

The main contributions of this paper are as follows: (1) a language-agnostic multimodal animation pipeline separating skeletal motion and facial blendshape into independent processing branches; (2) a functional web-deployed implementation with an operational animation asset library covering Kazakh and Russian sign language vocabularies; (3) a pilot expert evaluation combining classical GUI evaluation principles with sign language-specific linguistic criteria, yielding an overall mean score of 5.5/7 and with motion naturalness and transition consistency identified as the main areas for improvement.

## 2. Related Works

### 2.1. Methods for GUI Quality Evaluation

Existing assessments of GUI quality can be categorised into two distinct approaches: automated methods and human-involved methods. Human-involved methods are necessary because subjective perceptions of interface usability and effectiveness often cannot be fully automated. Human involvement is essential to identify real usability issues with the system [9]. Evaluations conducted by both end users and experts will facilitate the identification of real interaction difficulties.

Many different methods are used to evaluate graphical interfaces with user interaction. The most common methods involve user testing of the interface and subsequent evaluation using the Software Usability Scale (SUS), and more recently, the more modern Mobile Application Rating Scale (MARS). Both methods utilize specially designed surveys, which, after being completed by users, undergo relatively simple statistical processing.

Expert assessment methods involve experts in the field of usability and Human–Computer Interaction (HCI) who evaluate the interface according to established criteria and standards [10,11], using various techniques such as expert review, cognitive walkthrough, heuristic evaluation, guideline inspection, and others.

The specific needs of deaf and hard-of-hearing users require specialized visual assessment methods. These include the Funometer scale [12], which measures the user’s emotional engagement and mood changes. The Funometer visual scale (0–100) was developed to measure emotional engagement. It has been shown that technical imperfections in an avatar can cause a sharp drop in user mood by 23.89% [12].

When assessing objective indicators of sign language synthesis quality, visualisation accuracy is measured as the percentage of signs correctly understood by the user. The most important technical indicators concern the precision of fine motor skills of fingers and the smoothness of animation transitions. It is worth noting that even small deviations in the movement trajectory can significantly impair gesture comprehension [12]. To assess cognitive load and neural responses to grammatical errors in avatars, current research also utilizes biometric methods such as eye-tracking, electroencephalography (EEG), and functional magnetic resonance imaging (fMRI), which capture the intensity of user effort in processing visual information.

It is clear that, in addition to using technical metrics, the inclusion of measurable usability metrics is crucial. The SUS sets an average usability threshold of 68 points; scores exceeding 80.8 (class A) are considered excellent, while scores below 60 (class F) are considered critically low [13].

In the context of gesture avatars, the comprehension accuracy of synthesized, data-driven gestures reaches 74.87%, significantly exceeding the 56.12% of manual animation and even the 63.82% of novice human comprehension scores [14]. When designing such systems, it is necessary to consider non-manual markers (e.g., facial expressions and head position), which convey up to 70% of the linguistic meaning of a message [14].

The degree of similarity between a user and an avatar has also been shown to directly impact task performance. In the context of creative processes, a high degree of similarity has been shown to increase quantitative productivity (an index of 14.00 compared to 11.95 for “dissimilar” avatars) [15]. However, this increase in productivity is accompanied by a reduction in originality, with the originality index dropping to 1.84 (compared to 2.75 for avatars with low similarity) [15]. In the context of the Godspeed scale, anthropomorphism and animism of a real person are typically rated at 3.9 out of 5, while for virtual agents, these indices are significantly lower, ranging on average from 1.4 to 1.5 [15].

### 2.2. User-Centered Approaches and Avatars in HCI

In modern HCI, avatars are seen as part of a broader concept of user representations of virtual objects. That is, avatars serve as an artificial extension of the user’s physical body and allow the user to perform actions in a virtual environment. These representations allow the virtual world to be manipulated where physical impact is impossible. Manipulation of avatars occurs through motor commands, which generate a sense of agency (feeling of being the cause of the actions of a virtual object) [16].

The shift to intelligent Human–Computer Interaction (iHCI) is changing the design paradigm, where the machine as a tool is being replaced by a human–machine teaming model [17]. According to this source, modern human-oriented design iHCI is based on three key components. The technological component-focused on the recognition of the physiological, cognitive and emotional states of the user. The second component is related to the human factor and involves the development of models of user intent and cognitive-oriented interfaces, within which the user retains a decisive role and final control over the interaction process. The third component is an ethical component aimed at ensuring data confidentiality, transparency and accountability of the algorithms used.

The effectiveness of avatars in HCI is due to several cognitive phenomena. The first phenomenon, thanks to visual-motor synchronicity, the brain can perceive the virtual body as its own [16,18,19]. The second phenomenon is the process of psychological connection, when the user sees in the avatar the embodiment of their own identity, values, or idealized characteristics [18,20]. Finally, the Proteus effect [18]. For example, using athletic avatars in VR can reduce perceived physical effort and heart rate during exercise [18].

To strengthen the connection between the user and the system, avatar customization plays a key role. Customization enhances the sense of ownership of the virtual body and identification. Studies show that customized athletic avatars are more effective in reducing physiological responses to exercise (such as heart rate) than randomly assigned ones [18]. The high similarity of the avatar to the user increases involvement in the task, but in creative tasks, excessive similarity can limit the originality of ideas due to the activation of familiar thinking patterns [15].

It is important to take into account the specifics of different user groups in modern human-centric approaches. Avatars should provide means for self-expression through full-body models, customizable assistive technologies (e.g., wheelchairs, hearing aids) and the ability to control identity disclosure [21]. When designing avatars, for example, for Arabic culture, cultural markers (clothing, modesty) must be taken into account, which directly affects the level of trust and the intention to use the system [22]. Using avatars to express emotions and create an emotional connection is critical in health care applications, such as stroke rehabilitation [22].

In the context of human-centric design, the development of avatars for sign language moves from purely technical synthesis tasks to a deep account of the cognitive and linguistic characteristics of deaf and hard of hearing users. The sources emphasize that the key factor is Age Of Acquisition (AOA), namely users who learned sign language later experience greater cognitive load when processing syntax and need a slower pace and enhanced visual cues [23]. For such users, the avatar should provide signal redundancy and simplified syntactic structures to compensate for the difficulties of perception [23]. At the same time, native speakers from birth show a greater tolerance for technical imperfections, but more demanding of the naturalness of transitions between gestures [23].

Direct involvement of the Deaf Community at all stages of development is necessary to overcome systemic distrust of technologies developed without user inclusion. In the TAMSA (Technology Acceptance Model of Signing Avatars) model, the trust factor is as important as perceived utility and ease of use [24].

To increase acceptance of the system, avatars must reflect the user’s cultural identity through clothing and social norms. For example, the avatar “BuHamad” was designed with Qatari traditions in mind (the use of gutra and toba), which greatly improved its perception in the local community [12].

Traditional text questionnaires are often ineffective due to literacy barriers, so UCA involves the use of visual tools, such as sorting tasks with GIFs, visual rating scales, and Funometer-type tools to track emotional state [12].

Although the addition of emotional facial expressions (according to the Ekman system) does not always directly improve understanding of meaning, it is critical to the sense of naturalness and “humanity” of the avatar, which directly affects the long-term use of the system [14].

Thus, modern gesture synthesis systems should not simply reproduce hand movements, but adapt to the individual linguistic profile and cultural context of the user [23,24].

### 2.3. Evaluation of Sign Language Avatars

The evaluation of the quality of avatars, particularly those designed for translation into sign language, necessitates the involvement of experts and representatives of the target user group [23]. It is customary for studies to consider three factors in particular: the number of participants, their experience of sign language, and their professional profile. The evaluation process involves sign language speakers, professional sign language interpreters, and researchers specialising in the fields of linguistics and computer graphics [23,25].

The evaluation process involves the analysis of comprehensibility, naturalness, and correctness of gestures by experts, while the practical suitability of the system is determined through the involvement of end users. This combined approach is regarded as the most effective, as it facilitates both linguistic accuracy real-world usability of the avatar by end users [25].

Notwithstanding the active development of avatar technologies and their pervasive use in graphical user interfaces, the issue of assessing the quality of sign language avatars remains insufficiently formalized. In contradistinction to conventional GUI, where established usability assessment methodologies are employed, gesture avatars integrate multiple complex components, a visual interface, body and face animation, and the linguistic characteristics of sign language.

The existing corpus of research in the field of gesture avatars is focused on two main areas. Firstly, it addresses the technical correctness of gesture synthesis. Secondly, it encompasses aspects such as translation clarity, the naturalness of movements, the presence of non-manual components, user acceptance, and overall User eXperience (UX) [12,26]. However, the evaluation methodologies used across studies show significant variability depending on avatar type (e.g., rule-based, data-driven, motion capture) and participant profiles, which limits the comparability of results [27,28,29].

Despite the active development of avatar technologies and methods for evaluating graphical user interfaces, there is still no unified, formalised approach in the field of gesture avatars that combines avatar development and systematic quality evaluation.

## 3. Materials and Methods

### 3.1. SignBridge Avatar: System Architecture and Animation Pipeline

**Figure 1:**
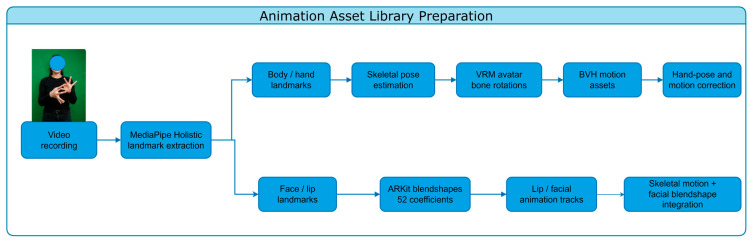
Stage 1: Animation Asset Library Preparation.

**Figure 2:**
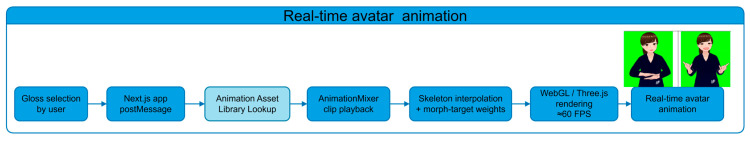
Stage 2: Real-Time Avatar Animation.

The SignBridge avatar forms the core of the proposed system, transforming the recorded movements of a professional sign language interpreter into a digital communication agent capable of reproducing sign language for deaf and hard-of-hearing users, as demonstrated here using the Kazakh and Russian languages [30]. Rather than serving as a decorative graphic element, the avatar functions as the primary component of the graphical user interface that enables information accessibility. The overall pipeline consists of two interrelated stages, illustrated in Figure 1 and Figure 2.

Video recordings of a professional sign language interpreter are processed using MediaPipe Holistic (version 0.5.1675471629 with Python/backend version 0.10.35), which extracts body, hand, and facial landmarks [31,32,33,34]. The extracted landmarks are split into two independent processing branches. The first branch processes body and hand landmarks: skeletal pose parameters are estimated, VRM avatar bone rotations are computed [6,35,36,37], BVH motion assets are generated, and hand-pose and motion correction is applied to refine wrist orientation and finger configuration The second branch processes face and lip landmarks: 52 ARKit blendshape coefficients are computed, from which facial and lip animation tracks are generated. The results of both branches are combined into a single animation asset, a skeletal animation with integrated facial morphs, which is stored in the library and linked to the corresponding gloss.

When the user initiates playback, the Next.js application passes the selected gloss via postMessage. The system retrieves the corresponding animation asset from the Animation Asset Library and triggers playback via AnimationMixer (built in Three.js version 0.169.0). Smooth transitions between glosses are achieved through interpolation of skeletal poses and blendshape weights. The final animation is rendered via WebGL/Three.js (version 0.169.0) at approximately 60 frames per second, ensuring real-time responsiveness.

#### 3.1.1. Definition of Input Data

The system receives input data consisting of *K* keypoints extracted from video recordings using MediaPipe Holistic:

(1) $$K=\left\{k_{i}\right\},\;\;k_{i}=(x_{i},y_{i},z_{i}),\;\;i=1,2,...,N$$

where N denotes the number of keypoints, while $x_{i},y_{i},z_{i}$ represent the coordinates of each *i* keypoint.

The input representation is organized into two complementary branches that reflect the dual nature of sign language. The first branch consists of body and hand landmarks, which are used to estimate skeletal pose parameters and compute VRM avatar bone rotations. The second branch consists of face and lip landmarks, which are used to generate ARKit blendshape coefficients for facial expression and lip articulation. Together, these branches cover both manual components and non-manual components, all of which are linguistically significant in sign language.

**Figure 3:**
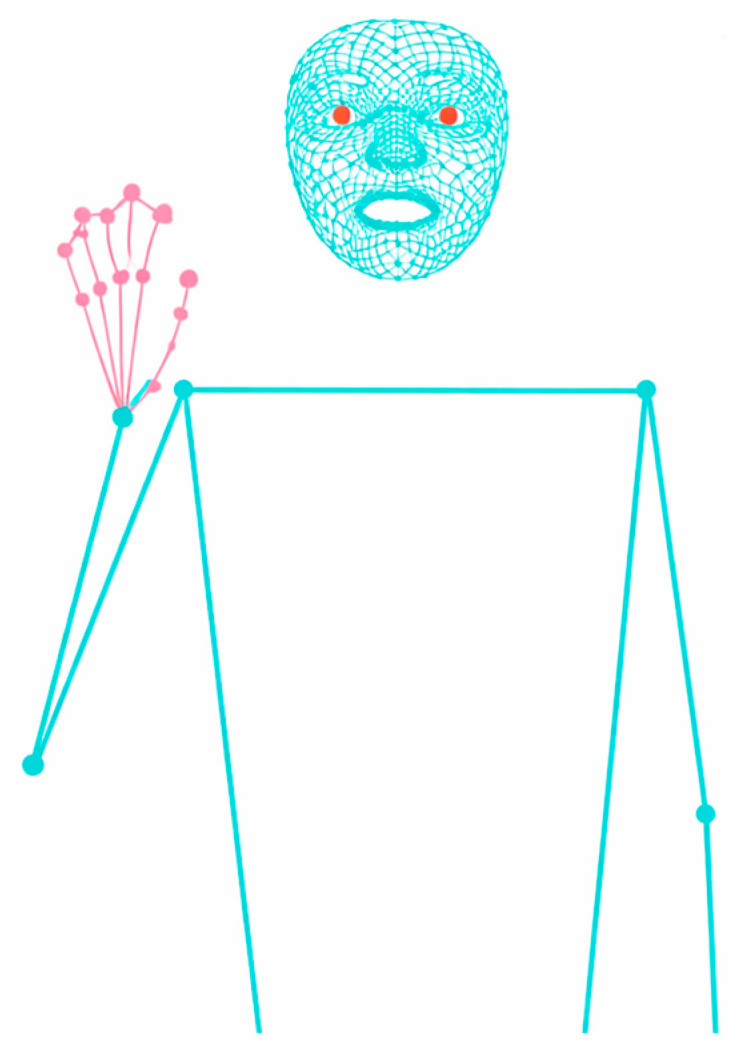
Example of MediaPipe landmarks extracted from sign-language video.

Unlike isolated coordinate points, the skeletal representation connects landmarks into anatomically meaningful joint pairs and converts them into bone vectors, local rotations, and motion parameters [31,32,33,34]. The keypoint representation therefore serves as an intermediate stage between raw video input and avatar-controllable animation, as illustrated in Figure 3.

#### 3.1.2. Core Mapping Functions

Blendshape parameters are defined as parametric representations of non-rigid surface deformations of the 3D avatar model that capture facial expressions, lip articulation, and fine muscle-driven movements that cannot be reproduced by skeletal transformations alone [38,39,40].

Each keypoint $k_{i}$ mapped to either skeletal transformation parameters $T_{j}$ or blendshape presets $B_{j}$:

(2) $$T_{j}=f_{T}\;(k_{i}),\;\;\;\;\;\;\;\;B_{j}=f_{B}\;(k_{i})$$

where $T_{j}\;$ denotes the set of skeletal transformation parameters (e.g., joint rotations and translations), $B_{j}$ represents ARKit blendshape activation parameters controlling non-rigid surface deformations of the avatar. The functions $f_{T},f_{B}$ are transformation functions corresponding to the structure of the VRM avatar.

The mapping process follows three sequential steps. First, detected landmarks are converted into an articulated skeletal representation, where anatomically connected keypoints form bone vectors and joint relationships. Next, these relationships are used to estimate local bone rotations and translations. Then, the calculated transfor-mations are applied to the corresponding bones of the VRM avatar, while face and lip landmarks are separately mapped to ARKit blendshape coefficients.

#### 3.1.3. Skeletal and Hand Animation

The reproduction of body and arm movements is facilitated by the utilisation of skeletal transformation parameters $R_{ij}$, which are determined by the relative position of the joints:

(3) $$R_{ij}\;=\;k_{j}\;-k_{i},\;\forall \;i,j\;\in C\;$$

where C represents the predefined set of joint connections defined by the avatar’s skeletal topology (i.e., the kinematic tree describing anatomical parent–child relationships between joints). Each element i,j ∈C corresponds to a valid bone in the skeleton, such as shoulder–elbow, elbow–wrist, wrist–finger, or interphalangeal finger joints. Transformations $T_{j}$ are calculated as follows:

(4) $$T_{j}=f_{R}\;\left(R_{ij}\right),$$

where $f_{R}$ describes the rotation and translation required to represent gestures and poses accurately.

Since sign language comprehension strongly depends on hand shape and wrist orientation, the generated motion is further refined through hand-pose and motion correction. This step adjusts finger configuration, wrist direction, and motion stability before the animation asset is stored in the library.

**Figure 4:**
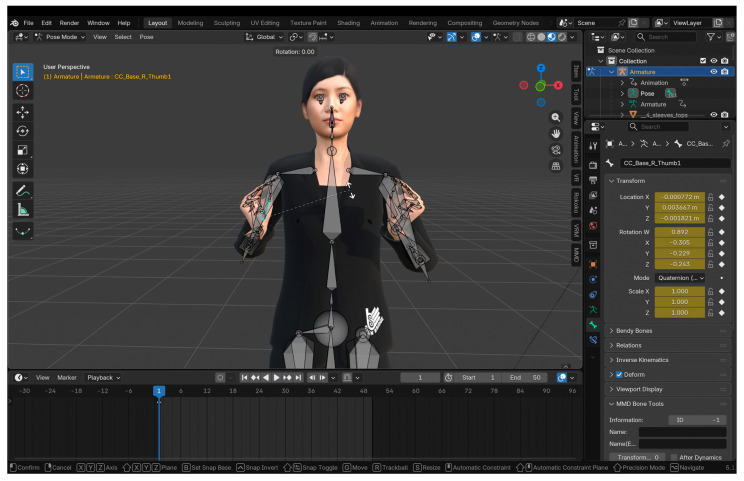
Application of skeletal motion parameters to the SignBridge Avatar armature.

The resulting skeletal motion can be stored as BVH motion assets during the preparation stage and then converted into AnimationClip, GLB, or JSON-compatible animation assets for web-based avatar playback. These assets contain keyframe tracks for the bones of the avatar skeleton and can be linked to individual glosses or phrase-level constructions in the SignBridge animation asset library, as illustrated in Figure 4.

#### 3.1.4. Facial Expression Synchronization

To reproduce facial expressions, blendshape coefficients E are used, which control the deformation parameters of the 3D model:

(5) $$E=(e_{1},e_{2},...,e_{M})$$

where M is the number of presets.

Each coefficient $e_{m}$ depends on the corresponding face or lip landmark:

(6) $$e_{m}=f_{E}\;(k_{i}),\;\forall \;i\;\in \;\mathrm{face and lip landmarks}$$

*f_E_* is a mapping function that converts geometric relationships between face and lip landmarks, such as inter-landmark distances, angles, and relative displacements, into normalized blendshape activation values.

In the proposed pipeline, face and lip landmarks are transformed into 52 ARKit blendshape coefficients that control non-rigid facial deformation, reproducing mouth opening, lip closure, lip rounding, jaw movement, eyebrow motion, and other non-manual components essential for natural sign language animation.

**Figure 5:**
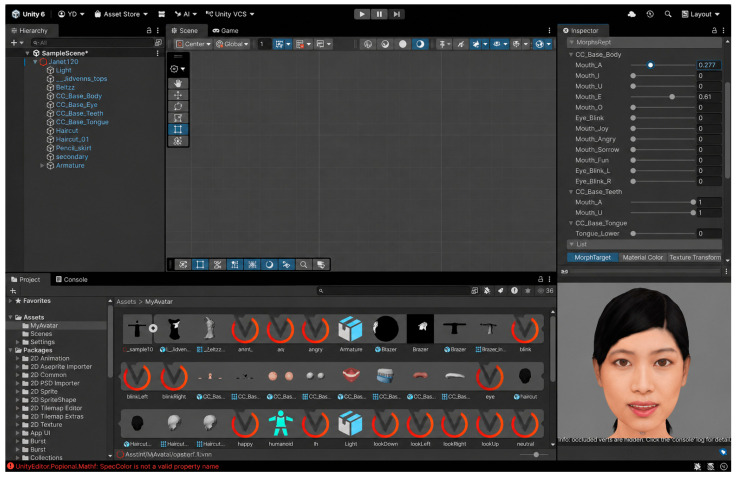
Facial expression and lip synchronization using ARKit blendshape parameters.

The computed blendshape coefficients are stored as facial animation tracks and synchronized with the skeletal motion from the first branch. As a result, manual gestures, facial expression, and lip articulation are combined into a single unified animation asset stored in the SignBridge Animation Asset Library, as illustrated in Figure 5.

#### 3.1.5. Smoothness and Interpolation

To prevent abrupt transitions between frames, interpolation is applied at the keypoint level. Linear interpolation is calculated as follows:

(7) $$k_{i}(t)=ak_{i}(t-1)\;+(1-a)\;k_{i}(\;t+1),$$

where a ∈[0,1] is the smoothing coefficient.

For smoother transitions between frames, spline interpolation is used:

(8) $$\;k_{i}(t)=\sum_{n=0}^{N}c_{n}B_{n}(t),$$

where $B_{n}(t)$ are the basis functions of the B-spline, and $c_{n}$ are the corresponding coefficients.

This approach reduces frame-to-frame jitter and improves the naturalness of the generated motion. During avatar playback, interpolation is also applied to skeletal keyframes and morph-target weights. The bones of the body, arms, hands, and fingers are smoothly interpolated between keyframes, while facial blendshape coefficients are updated synchronously with the skeletal motion.

The final animation of the avatar $A_{t}$ is generated by integrating the keypoint data, bone transformation parameters, and blendshape coefficients. The overall state of the avatar at time $A_{t}$ is defined as follows:

(9) $$A_{t}=\left\{T_{j}\left(t\right),\;B_{j}\left(t\right),E\left(t\right)\right\},$$

where $T_{j}(t)$ represents the skeletal transformation parameters responsible for body, shoulder, arm, and head positions and orientations; $B_{j}(t)$ denotes the hand and finger motion parameters; E(t) includes the ARKit blendshape coefficients for facial expression and lip articulation. For each animation frame t, the avatar engine retrieves the corresponding values of $T_{j}\left(t\right),\;B_{j}\left(t\right)$ and $E\left(t\right)$ from the selected AnimationClip. The skeletal parameters $T_{j}\left(t\right),\;B_{j}\left(t\right)$ are applied to the corresponding bones of the avatar rig, while $E\left(t\right)$ updates the morph-target weights of the facial mesh. Therefore, Equation (9) defines the frame-level state of the avatar and specifies how skeletal motion, hand articulation, facial expression, and lip movement are synchronized during playback.

At each frame, the avatar animation engine updates the state defined by Equation (9): AnimationMixer advances the skeletal keyframes and morph-target weights, and the WebGL/Three.js renderer draws the result in the browser.

The two-stage design—offline asset preparation and online playback—means that the heavy processing happens once, during recording and correction, while the runtime component stays light enough to run in any standard web browser without specialised hardware. In practice, this shifts the system from fixed pre-recorded sign videos toward flexible, gloss-driven animation that can be assembled and played back on demand.

### 3.2. Evaluation Methods

The present evaluation is a pilot study. Professional sign language interpreters were chosen as evaluators because they can assess gesture accuracy, spatial articulation, and non-manual components with linguistic precision—which is exactly what is needed at this early stage to catch technical problems before wider testing. Five experts is a typical panel size for this kind of pilot: HCI research has shown that three to five evaluators are enough to surface most critical usability issues [7,8]. Involvement of the Deaf Community as end users is planned as the next stage of validation.

**Figure 6:**
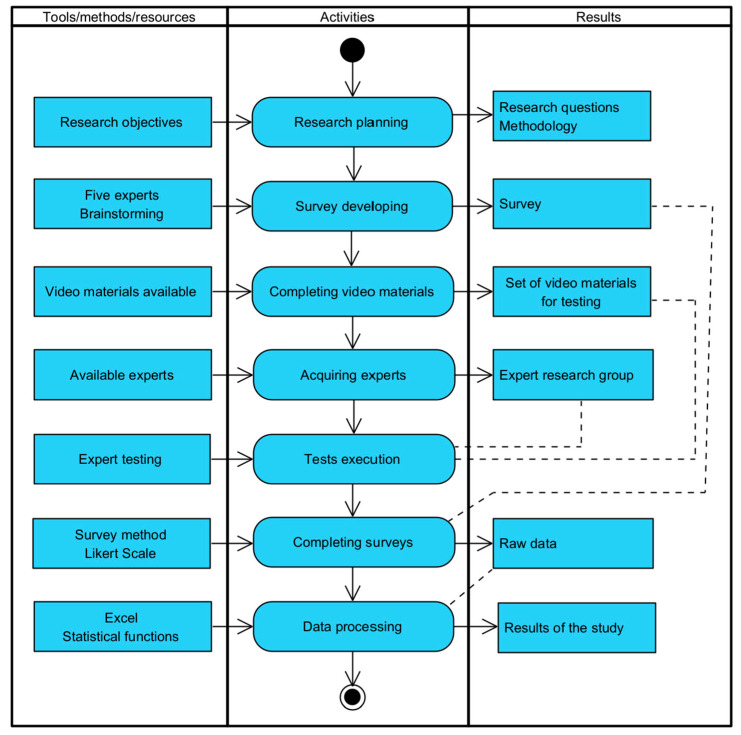
Research workflow.

The assessment of SignBridge Avatar quality was systematically structured into several phases: the definition of research objectives, the development of assessment criteria through expert brainstorming, and the execution of evaluation procedures, as illustrated in Figure 6.

Data collection and expertise were carried out in collaboration with several organizations, such as Institute of Information and Computational Technologies (Almaty) [41], the L.N. Eurasian National University Gumilyov (Astana) [42], Kazakh Society of the Deaf [43] and SignBridge LLP [30].

### 3.3. Experts and Brainstorming Methods Implementation

In the initial phase of developing the evaluation methodology, expert brainstorming was conducted [7,8]. The brainstorming process was organised based on the practical experience of the expert group. The expert group comprised five professional sign language interpreters (1 male and 4 female) with an average age of 41 years and an average of 22 years of active sign language practice, all engaged in daily interpretation work. The discussions were conducted in a structured format, focusing on aspects such as the clarity of gestures, the precision of movement, spatial articulation, the synchronization of facial expressions and hand movements, as well as the overall appearance and suitability of the avatar.

The brainstorming session yielded several key attributes, including the avatar system’s appearance, color harmony, synchronization of facial expressions and hand gestures, and overall usability [44]. These attributes were identified through expert discussion, thereby establishing the theoretical foundation for the subsequent questionnaire design and evaluation categories.

### 3.4. Developed Survey

In accordance with the outcomes of the expert brainstorming session, the quality of avatar animation was quantitatively evaluated using a Likert scale [6,10]. This scale is extensively utilised within the domain of HCI research to quantitatively process expert subjective assessments. The scale ranged from 1 (very low/strongly disagree) to 7 (very high/strongly agree). A structured questionnaire was developed to cover various aspects of sign language visualization [27].

The survey consisted of the following five thematic groups (Table 1).

The questionnaire was developed based on the quality attributes identified during the expert brainstorming session described in Section 3.3 [13,22,24]. The use of the structured questionnaire is justified by the need for quantitative analysis of subjective expert evaluations in the field of HCI [13].

Since the avatar in this system acts as not just as a visual element, but a communicative agent, the questionnaire was designed to combine the classical principles of GUI evaluation with the linguistic features of sign language [22,23]. This allows the evaluation of both the technical correctness of gesture synthesis and the overall system perception by the user (User Experience, UX) [22,23].

Each question in these groups was formulated in such a way that experts could assess specific aspects of the animation in detail, facial coordination, or the naturalness of pauses at sentence boundaries. This comprehensive approach to the questionnaire structure provides a solid basis for evaluating the quality of visualisation, both at the word level and in a continuous dynamic context.

### 3.5. General Rules for Selecting Video for Research

**Figure 7:**
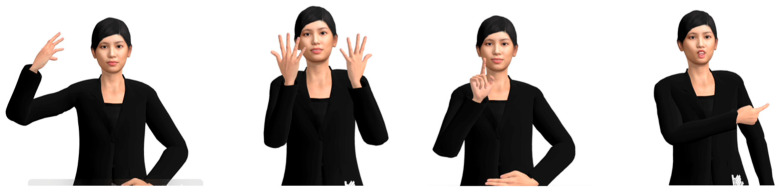
Examples of individual lexical signs. From left to right: “Smart”, “Light”, “Tasty”, “Strong” in KSL.

**Figure 8:**
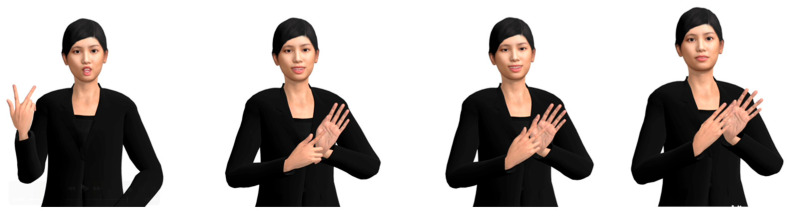
Examples of numeral signs. From left to right: “3”, “7”, “8”, ”9”.

**Figure 9:**
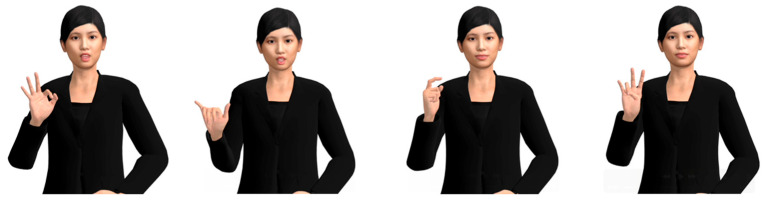
Examples of finger spelling (dactyl alphabet). From left to right: “O Y X H”.

The evaluation dataset was compiled from real-world multimedia content processed through the full SignBridge pipeline, including common individual lexical signs (Figure 7), signs for numerals (Figure 8), and the dactyl alphabet (Figure 9).

Each source video was processed through the full SignBridge pipeline: speech or text was first extracted, then converted into a gloss sequence, after which the matching animation assets were pulled from the SignBridge Animation Asset Library and rendered as sign language animation. The resulting avatar videos were compiled as the evaluation dataset.

The SignBridge Animation Asset Library currently comprises approximately 2000 gloss-level assets covering Kazakh Sign Language (KSL) and Russian Sign Language (RSL) vocabularies [30]. New assets are added and corrected manually on an ongoing basis as the vocabulary grows.

### 3.6. Set of Selected Video for Testing

**Figure 10:**
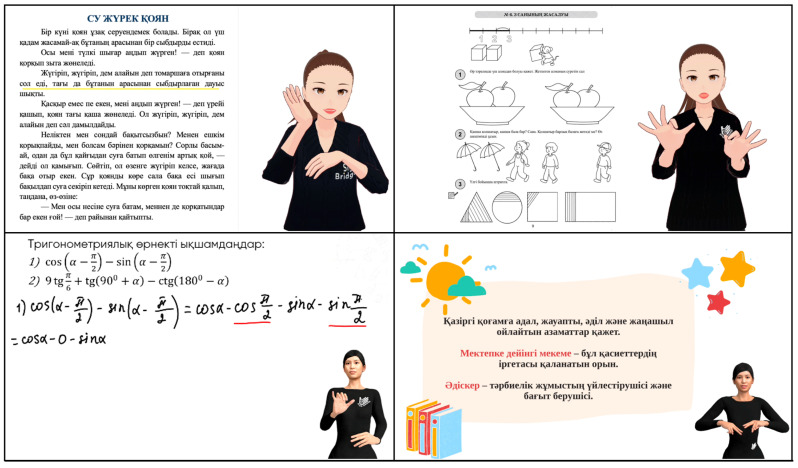
Examples of video materials selected for the testing.

Before the assessment, all materials underwent mandatory technical verification, including synchronisation of avatar animation with the source audio track, video alignment, adjustment of gesture speed for comfortable perception, and verification of the clarity of the avatar’s hands and face. Transitions between gestures at sentence boundaries were refined to ensure natural continuity (Figure 10). Working dynamic avatar examples (male: AIBeck and female: AINaz) are presented at: https://signbridge.kz/ (accessed on 10 January 2026), as well as sample avatar videos used for research.

The selected materials had a total volume of approximately 5871 MB and a presentation time of 58 min, covering the following domains: cartoons, educational materials ranging from preschool to secondary school level, short stories and fairy tales, seminar and conference presentations, interactive illustrative content, and sample translations of programme segments. This heterogeneous composition was designed to ensure broad domain coverage and robust evaluation across diverse content types.

### 3.7. Research Implementation

**Figure 11:**
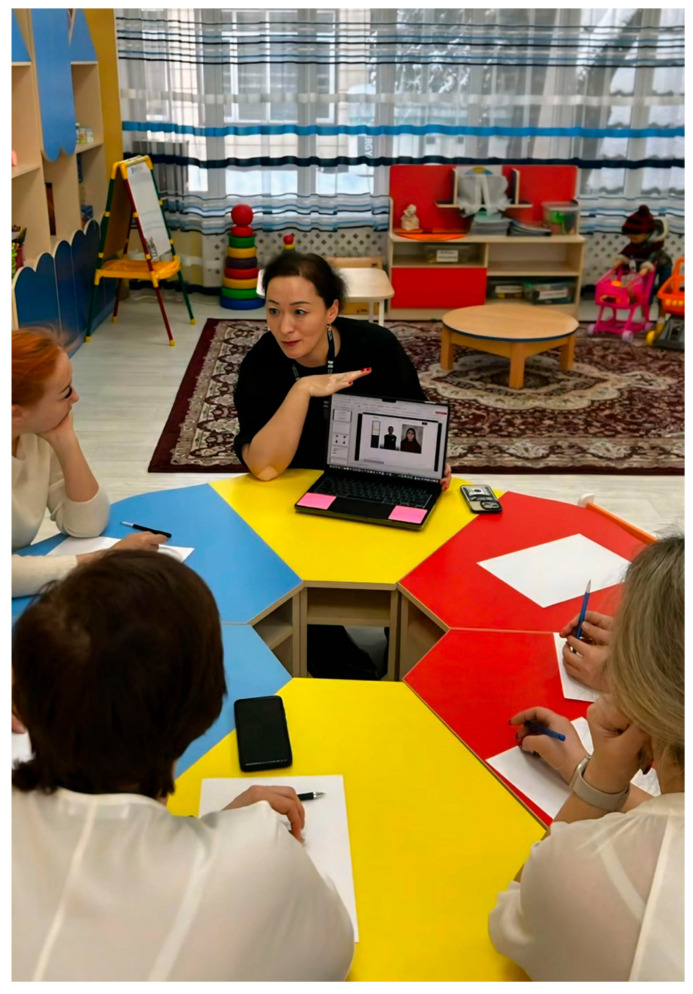
The process of conducting the research with the experts (the face of one of the co-authors is visible in the photo).

Following the briefing and consent procedure, experts independently viewed the selected video materials (Figure 11) and completed the evaluation surveys. Raw data obtained from the surveys were subjected to statistical processing, including calculation of mean values, standard deviations, and Cronbach’s alpha coefficients.

Raw data obtained from the surveys subjected to statistical processing, resulting in the final research outcomes (Figure 6).

## 4. Results

### Overview of Expert Evaluation Results

The evaluation was conducted by the expert group described in Section 3.3. The ratings provided by the experts, evaluated using a 7-point Likert scale, demonstrated an overall mean average of 5.5 (standard deviation: 1.6) across all criteria, thereby signifying that the system’s overall performance quality is deemed to be satisfactory (see Table 2).

Additionally, statistical analysis included the calculation of mean values (MV), standard deviations (SD), and Cronbach’s alpha coefficients for each group of questions (Table 2).

**Figure 12:**
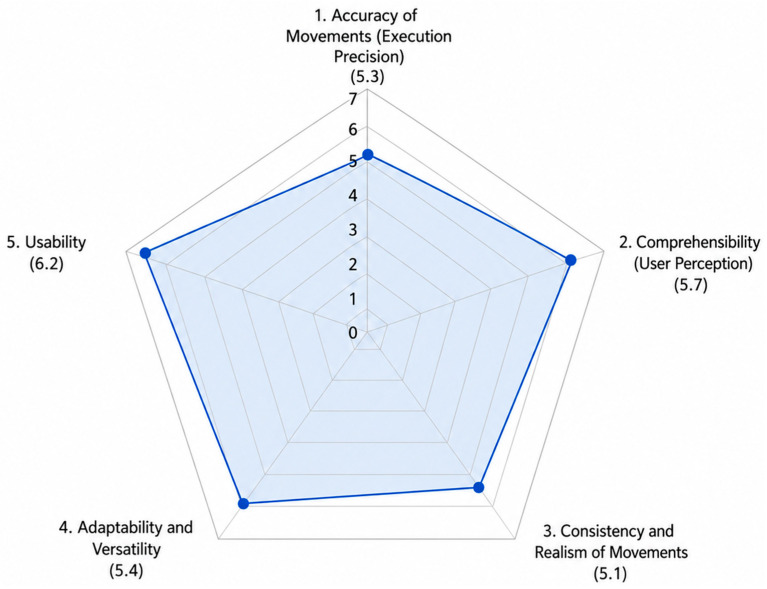
Radar chart of mean expert evaluation scores by assessment group.

To provide a clearer group-level comparison, the mean scores for the five assessment groups are presented in Figure 12.

Figure 12 shows that the highest score was obtained for Usability (MV = 6.2, SD = 1.3), followed by Comprehensibility (MV = 5.7, SD = 1.4). The lowest score was observed for Consistency and Realism of Movements (MV = 5.1, SD = 1.8), indicating that movement smoothness, transition consistency, and natural signing rhythm remain the main aspects requiring further improvement. The overall mean score across all criteria was 5.5 (SD = 1.6).

## 5. Discussions

Five professional sign language interpreters evaluated the SignBridge Avatar as part of a pilot study. Their ratings averaged 5.5 out of 7 across all criteria, a score that points to practical potential while leaving clear room for improvement. Five evaluators is a standard panel size for this type of pilot, as three to five experts are generally enough to surface the most critical usability issues [7,8].

To put this number in context, the System Usability Scale sets 68/100, roughly 4.76/7, as the threshold above which a system is considered acceptable for practical use [13]. The SignBridge Avatar exceeded this threshold, and the two highest-scoring areas, usability (6.2/7) and comprehensibility (5.7/7), are precisely the ones that matter most for an avatar acting as a communicative interface element. That said, a stronger claim would require a larger group of evaluators and direct involvement of deaf users, which is the planned next step.

A detailed comparison of evaluation results reveals the following pattern. The usability of the avatar achieved a mean score of 6.2, with appearance suitability rated at 5.6, colours and visual style at 5.8, design and interface at 6.4, and external appearance at 6.2.

Within the comprehensibility metric, the adequacy of gesture presentation speed was identified as the highest individual indicator, reaching 6.4. Ease of understanding gesture meaning was rated at 5.8, gesture clarity at 5.6, and the execution of gesture sequences at 5.0.

The mean accuracy of movements was 5.3, with adherence to standards at 5.6, spatial parameters at 5.2, and finger and wrist movements also at 5.2, while hand shape and overall movement accuracy were comparatively lower at 5.0.

With regard to adaptability and versatility, the performance of complex sentences was rated at 5.0, letters and numbers at 5.4, spatial-temporal parameters 5.2, while hand shape and overall movement accuracy were lower at 5.0.

The lowest mean score was observed in the consistency and realism metric (5.1). Specifically, smoothness and naturalness of movement were rated at 5.2, while abrupt transitions and gesture speed realism both received 4.8, indicating limitations in temporal coherence between glosses.

The most noticeable problems appeared at gloss boundaries, where the transition between two consecutive signs occasionally looked unnatural. This is a side effect of storing each gloss as a separate animation clip: when clips are joined, continuity relies entirely on interpolation, and the result is not always smooth. Sentences with rapid spatial shifts and finger-spelling sequences where adjacent signs share similar handshapes were the hardest cases for the system. These observations match the lower scores for gesture sequence execution (5.0) and motion smoothness (5.2), and they point directly to where development effort should go next.

Future versions of the pipeline would benefit from more structured monitoring of motion consistency, rendering responsiveness, and error propagation between modules, drawing on hybrid AI-based validation approaches that have shown promise in similar complex systems [45].

With regard to extant scientific works, although Chakladar et al. [46] demonstrated the feasibility of continuous gesture generation, their evaluation results were limited to a single language, and the issue of multilingual adaptation was not fully addressed In contrast, the current results indicate broader adaptability (5.4) across linguistic structures.

As highlighted by Dimou and et al. [27] user perception and naturalness remain critical factors in avatar-based sign language communication. The relatively lower scores in motion consistency observed in this study confirm that this challenge persists. However, the high usability (6.2) and strong comprehensibility (5.7) indicate that the system achieves a favorable level of user acceptance.

Compared to the systems proposed by Morillas-Espejo et al. which are accessible but limited in handling complex linguistic structures, the present approach demonstrates improved capability in executing complex sentences (5.2) and representing spatial-temporal markers (5.6).

Furthermore, although end-to-end neural approaches such as those described by De Coster et al. [47], achieve high realism, they require large datasets and significant computational resources. In contrast, the proposed method employs a lightweight and modular architecture, achieving a competitive overall performance (5.5) while remaining suitable for web-based deployment.

Regarding statistical robustness, given the pilot nature of the study and the small sample size (n = 5), standard significance tests and effect size analyses were not performed, as they would not yield statistically meaningful results at this sample size. Internal consistency was assessed through Cronbach’s alpha, which exceeded 0.9 for four of the five question groups, indicating strong agreement in evaluator rating patterns, while the value of 0.81 for the Comprehensibility group reflects good and acceptable reliability. Inter-rater variability is reflected in the reported standard deviations. Expanding the evaluator pool in future studies will enable fuller statistical analysis including confidence intervals and inter-rater agreement coefficients.

Overall, the results confirm the effectiveness of the proposed system for bilingual sign language visualisation, particularly in terms of usability and comprehensibility. Motion naturalness and transition consistency at gloss boundaries remain the primary directions for technical improvement. These findings underscore the importance of balancing pipeline automation with user-centred evaluation in the development of inclusive, web-based sign language avatar systems.

## 6. Conclusions

In the context of linguistic globalization, historical language transformations, and increasing demands for bilingualism in professional environments, the need for effective digital solutions that ensure information accessibility for users with hearing impairments has become apparent. In the context of multilingual and inclusive digital communication, the issue of evaluating a sign language avatar as both a GUI element and a communication agent was raised.

The research presents a functional, web-deployed bilingual sign language avatar system with a validated two-stage animation pipeline and an operational animation asset library of approximately 2000 gloss-level assets. The quality of the system was assessed through a pilot expert evaluation combining classical GUI evaluation principles with the linguistic features specific to sign language. The high scores for clarity and usability in the expert evaluation demonstrate the avatar’s effectiveness as an interface element. The analysis identified the naturalness of the movements and the consistency of transitions between gestures as aspects requiring further improvement. The conducted research is characterized by a high level of reliability (internal consistency) of the results (Cronbach’s alpha coefficient > 0.8), which ensures their credibility.

The results show that a modular, asset-library-based architecture can deliver sign language avatar functionality in a web browser without specialised hardware, and at a quality level that clears standard usability thresholds. The approach is demonstrated here for Kazakh and Russian, but the underlying pipeline is not language-specific and can be adapted to other sign languages.

## 7. Future Works

The next stage of development focuses on two main directions. On the system side, the gloss vocabulary will be expanded, and the manual blendshape correction process will be gradually supported by automated quality checks. On the evaluation side, a user study with deaf and hard-of-hearing participants is planned, where the current expert survey will be supplemented with objective perceptual metrics, including motion smoothness, temporal coherence, gesture recognition accuracy, and facial synchronisation error. The evaluation will also be extended with biometric tools such as eye-tracking and EEG. Together, these steps aim to move the system toward practical deployment.

## Figures and Tables

**Table 1:** Developed questionnaire for research.

Groups of Questions	Questions
1. Accuracy of Movements (Execution Precision)	1.1. How accurately does the avatar convey hand shapes and movements?
	1.2. Do the performed sign gestures conform to the accepted sign language standards?
	1.3. Are the spatial parameters of the movements correctly represented?
	1.4. How accurately are finger and wrist movements displayed?
2. Comprehensibility (User Perception)	2.1. How easy is it to understand the meaning of the gestures performed by the avatar?
	2.2. How clear and distinguishable are the gestures?
	2.3. Is the speed of gesture presentation sufficient for comprehension?
	2.4. How correctly is the sequence of gestures executed?
3. Consistency and Realism of Movements	3.1. How smooth and natural are the avatar’s movements?
	3.2. Are there any abrupt or unnatural transitions between gestures?
	3.3. Does the speed of gestures correspond to the natural pace of sign language?
4. Adaptability and Versatility	4.1. How well does the avatar execute complex sentences?
	4.2. Does the avatar correctly display individual letters and numbers?
	4.3. Can the avatar accurately represent gestures containing spatial and temporal markers?
	4.4. How well does the avatar adapt to different linguistic structures?
5. Usability	5.1. How suitable is the avatar’s appearance and image for perception?
	5.2. Are the avatar’s colors and visual style pleasing to the eye?
	5.3. Rate the design and interface
	5.4. Rate the avatar’s external appearance.

**Table 2:** Expert Evaluation Results of the Survey.

Groups of Questions	Questions	MV/SD for Questions	MV/SD for Groups Cronbach’s Alpha
1. Accuracy of Movements (Execution Precision)	1.1 How accurately does the avatar convey hand shapes and movements?	5.0/2.1	5.3/1.7 0.98
	1.2 Do the performed sign gestures conform to the accepted sign language standards?	5.6/1.3	
	1.3 Are the spatial parameters of the movements correctly represented?	5.2/2.0	
	1.4 How accurately are finger and wrist movements displayed?	5.2/1.5	
2. Comprehensibility (User Perception)	2.1 How easy is it to understand the meaning of the gestures performed by the avatar?	5.8/1.1	5.7/1.4 0.81
	2.2 How clear and distinguishable are the gestures?	5.6/1.5	
	2.3 Is the speed of gesture presentation sufficient for comprehension?	6.4/0.9	
	2.4 How correctly is the sequence of gestures executed?	5.0/1.9	
3. Consistency and Realism of Movements	3.1 How smooth and natural are the avatar’s movements?	5.2/1.9	5.1/1.8 0.98
	3.2 Are there any abrupt or unnatural transitions between gestures?	4.8/1.9	
	3.3 Does the speed of gestures correspond to the natural pace of sign language?	4.8/2.0	
4. Adaptability and Versatility	4.1 How well does the avatar execute complex sentences?	5.2/2.0	5.4/1.6 0.97
	4.2 Does the avatar correctly display individual letters and numbers?	5.4/2.1	
	4.3 Can the avatar accurately represent gestures containing spatial and temporal markers?	5.6/1.7	
	4.4 How well does the avatar adapt to different linguistic structures?	5.6/1.1	
5. Usability	5.1 How suitable is the avatar’s appearance and image for perception?	5.6/2.2	6.2/1.3 0.95
	5.2 Are the avatar’s colors and visual style pleasing to the eye?	5.8/2.2	
	5.3 Rate the design and interface.	6.4/1.3	
	5.4 Rate the avatar’s external appearance.	6.2/1.3	
Overall	5.5/1.6		

## Data Availability

The original contributions presented in the study are included in the article; further inquiries can be directed to the corresponding author.
